# Supplementary material for: Sociological dimensions of marital satisfaction in Romania
Source: PLoS One. 2020 Aug 20;15(8):e0237923. doi: 10.1371/journal.pone.0237923 (PMC7446780; doi:10.1371/journal.pone.0237923)
Supplement: S1 File — (DOCX) [file pone.0237923.s001.docx]

**S1 File. English questionnaire.**

**SOCIOLOGICAL QUESTIONNAIRE (only analyzed items)**

**Q5.** To what extent do you agree with partners living together before marriage?

1. to a very small extent

2. to a small extent

3. to some extent

4. to a large extent

5. to a great extent

6. don't know / prefer not to answer

**Q8.** To what extent do you agree with the freely agreed union?

1. to a very great extent

2. to a great extent

3. to some extent

4. to a small extent

5. to a very small extent

**Q11.** To what extent do you consider that, over the course of life, material conditions can keep a couple together?

1. to a very large extent

2. to a great extent

3. to some extent

4. to a small extent

5. to a very small extent

**Q17.** In general, which phrase best describes your relationships with your parents or legal guardians?

1. we got on very well

2. we got on well

3. indifference

4. we got on bad

5. we got on very bad

**Q18.** In general, what was the relationship between your parents like?

1. very good

2. good

3. indifference

4. tense

5. very tense

**Q19.** Personally, to what extent do you agree with sexual relations before marriage?

1. fully agree

2. partially agree

3. undecided

4. somewhat disagree

5. totally disagree

**Q24.** Do you consider that faith in God acts to reduce moral decline in the family, community and society in general?

1. yes

2. no

3. don't know

How long have you been married / in your current relationship OR state the duration of your last serious relationship: _______

Do you have children? Yes / No

**Dyadic Adjustment Scale**

Most persons have disagreements in their relationships. Please indicate below the approximate extent of agreement or disagreement between you and your partner for each item on the following list (Always Agree, Almost Always Agree, Occasionally Disagree, Almost Always Disagree, Always Disagree)

| **I1** | Handling family finances |  |
| --- | --- | --- |
| **I2** | Matters of recreation |  |
| **I3** | Religious matters |  |
| **I4** | Demonstrations of affection |  |
| **I5** | Friends |  |
| **I6** | Sex relations |  |
| **I7** | Conventionality (correct or proper behavior) |  |
| **I8** | Philosophy of life |  |
| **I9** | Ways of dealing with parents or in-laws |  |
| **I10** | Aims, goals, and things believed important |  |
| **I11** | Amount of time spent together |  |
| **I12** | Making major decisions |  |
| **I13** | Household tasks |  |
| **I14** | Leisure time interests and activities |  |
| **I15** | Career decisions |  |

How often… (all the time, most of the time, more often than not, occasionally, rarely, never)

| **I16** | How often do you discuss or have you considered divorce, separation or terminating your relationship? |  |
| --- | --- | --- |
| **I17** | How often do you or your mate leave the house after a fight? |  |
| **I18** | In general, how often do you think that things between you and your partner are going well? |  |
| **I19** | Do you confide in your mate? |  |
| **I20** | Do you ever regret that you married? (or lived together) |  |
| **I21** | How often do you and your partner quarrel? |  |
| **I22** | How often do you and your mate “get on each other’s nerves?” |  |

**I23.** Do you kiss your mate?

| Every Day | Almost Every Day | Occasionally | Rarely | Never |
| --- | --- | --- | --- | --- |
| 4 | 3 | 2 | 1 | 0 |

**I24.** Do you and your mate engage in outside interests together?

| All of them | Most of them | Some of them | Very few of them | None of them |
| --- | --- | --- | --- | --- |
| 4 | 3 | 2 | 1 | 0 |

How often would you say the following events occur between you and your mate? (Never, Less than once a month, Once or twice a month, Once a day, More often)

| **I25** | Have a stimulating exchange of ideas |  |
| --- | --- | --- |
| **I26** | Laugh together |  |
| **I27** | Calmly discuss something |  |
| **I28** | Work together on a project |  |

These are some things about which couples sometimes agree and sometime disagree. Indicate if either item below caused differences of opinion or were problems in your relationship during the past few weeks. (Check Yes or No)

| **I29** | Yes | No | Being too tired for sex. |
| --- | --- | --- | --- |
| **I30** | Yes | No | Not showing love. |

**I31.** The circles on the following line represent different degrees of happiness in your relationship. The middle point, “happy,” represents the degree of happiness of most relationships. Please fill in the circle which best describes the degree of happiness, all things considered, of your relationship.

| 0 | 1 | 2 | 3 | 4 | 5 | 6 |
| --- | --- | --- | --- | --- | --- | --- |
| Extremely Unhappy | Fairly Unhappy | A Little Unhappy | Happy | Very Happy | Extremely Happy | Perfect |

I32. Which of the following statements best describes how you feel about the future of your relationship?

1. I want desperately for my relationship to succeed, and would go to almost any length to see that it does.

2. I want very much for my relationship to succeed, and will do all I can to see that it does.

3. I want very much for my relationship to succeed, and will do my fair share to see that it does.

4. It would be nice if my relationship succeeded, but I can’t do much more than I am doing now to help it succeed.

5. It would be nice if it succeeded, but I refuse to do any more than I am doing now to keep the relationship going.

6. My relationship can never succeed, and there is no more that I can do to keep the relationship going.
