# Supplementary material for: Sociological dimensions of marital satisfaction in Romania
Source: PLoS One. 2020 Aug 20;15(8):e0237923. doi: 10.1371/journal.pone.0237923 (PMC7446780; doi:10.1371/journal.pone.0237923)
Supplement: S2 File — (DOCX) [file pone.0237923.s002.docx]

**S2 File. Romanian questionnaire.**

**Chestionar sociologic (doar itemii analizați)**

**Q5.** În ce măsură sunteți de acord ca partenerii să conviețuiască împreună, înainte de căsătorie?

1. în foarte mică măsură

2. în mică măsură

3. într-o măsură potrivită

4. în mare măsură

5. în foarte mare măsură

6. NȘ/NR

**Q8.** În ce măsură sunteți de acord cu uniunea liber consimțită ?

1. foarte mare măsură

2. mare măsură

3. într-o măsură potrivită

4. în mică măsură

5. în foarte mică măsură

**Q11.** În ce măsură considerați că, de-a lungul vieţii, condițiile materiale pot să menţină un cuplul?

1. foarte mare măsură

2. mare măsură

3. într-o măsură potrivită

4. în mică măsură

5. în foarte mică măsură

**Q17.** În general, care au fost relaţiile dumneavoastră cu părinții sau tutorii legali?

1. foarte bune

2. bune

3. indiferență

4. tensionate

5. foarte tensionate

**Q18.** În general, care au fost relaţiile dintre părinții dumneavoastră ?

1. foarte bune

2. bune

3. indiferență

4. tensionate

5. foarte tensionate

**Q19.** Dvs. personal, în ce măsură sunteți de acord cu relaţiile sexuale înainte de căsătorie?

1. cu totul de acord

2. parțial de acord

3. indecis

4. parțial dezacord

5. cu totul dezacord

**Q24.** Considerați că credința în Dumnezeu este un atenuator al degradării morale în familie, comunitate și societate în general?

1.da

2. nu

3. nu ştiu

De cât timp sunteți căsătorit/în relația actuală/durata ultimei relații serioase: _______

Aveți copii? Da Nu

**Chestionar pentru evaluarea satisfacţiei maritale**

Itemii de mai jos se referă la relaţia cu soţul / soţia (partenerul/partenera) dumneavoastră. Vă rugăm să indicaţi mai jos gradul de acord sau dezacord dintre dumneavoastră şi partenerul dumneavoastră pentru fiecare item în parte notându-le astfel:

*5 – Întotdeauna de acord*

*4 – Aproape întotdeauna de acord*

*3 – Dezacord ocazional*

*2 – Aproape întotdeauna dezacord*

*1 – Întotdeauna dezacord*

| **I1** | Organizarea finanțelor de familie |  |
| --- | --- | --- |
| **I2** | Recreerea în timpul liber |  |
| **I3** | Probleme religioase |  |
| **I4** | Demonstrarea afecţiunii |  |
| **I5** | Prieteni |  |
| **I6** | Relaţii sexuale |  |
| **I7** | Convenţionalitatea (comportamentul corect) |  |
| **I8** | Filosofia vieţii |  |
| **I9** | Modalităţi de a trata rudele |  |
| **I10** | Obiective, scopuri şi lucrări considerate importante |  |
| **I11** | Timpul liber petrecut împreună |  |
| **I12** | Luarea unor decizii majore |  |
| **I13** | Sarcini casnice |  |
| **I14** | Interese pentru petrecerea timpului liber |  |
| **I15** | Decizii legate de cariera profesională |  |

Vă rog să indicaţi cât de frecvent vi se întâmplă dumneavoastră şi partenerului dumneavoastră următoarele:

*1 – întotdeauna*

*2 – aproape tot timpul*

*3 – des*

*4 – ocazional*

*5 – rar*

*6 – niciodată*

| **I16** | Cât de des aţi discutat sau aţi luat în considerare divorţul, separarea sau terminarea relaţiei? |  |
| --- | --- | --- |
| **I17** | Cât de des dumneavoastră sau partenerul dumneavoastră plecaţi de acasă după o ceartă? |  |
| **I18** | În general, cât de des gândiți că lucrurile între dumneavoastră şi partenerul dumneavoastră merg bine? |  |
| **I19** | Aveţi încredere în partenerul dumneavoastră? |  |
| **I20** | Regretaţi vreodată că v-aţi căsătorit (sau că aţi trăit împreună)? |  |
| **I21** | Cât de des dumneavoastră şi pertenerul dumneavoastră vă certaţi? |  |
| **I22** | Cât de des vă călcaţi pe nervi unul pe altul? |  |

**I23.** Vă sărutaţi partenerul?

| În fiecare zi | Aproape în fiecare zi | Ocazional | Rar | Niciodată |
| --- | --- | --- | --- | --- |
| 4 | 3 | 2 | 1 | 0 |

**I24.** Dumneavoastră şi partenerul dumneavoastră vă angajaţi în satisfacerea intereselor comune împreună?

| Toate | Majoritatea | Unele | Puţine | Niciunul |
| --- | --- | --- | --- | --- |
| 4 | 3 | 2 | 1 | 0 |

Cât de des vi se întâmplă următoarele evenimente dumneavoastră şi partenerului dumneavoastră?

*1 – Niciodată*

*2 – Mai puţin de o dată pe lună*

*3 – O dată sau de mai multe ori pe lună*

*4 – O dată pe zi*

*5 – Mai des (de o dată pe zi)*

| **I25** | Aveţi schimburi simultane de idei |  |
| --- | --- | --- |
| **I26** | Râdeţi împreună |  |
| **I27** | Discutați calm ceva |  |
| **I28** | Lucraţi împreună la un proiect |  |

Acestea sunt unele lucruri cu care cuplurile uneori sunt de acord, iar alteori nu . Indicaţi care dintre itemii de mai jos v-au cauzat diferenţe de opinie sau probleme în relaţia dumneavoastră, în ultimele săptămâni (Încercuiţi DA sau NU)

| **I29** | DA | NU | Aţi fost prea obosiţi pentru a face sex. |
| --- | --- | --- | --- |
| **I30** | DA | NU | Nu prea aţi arătat iubire. |

**I31.** Numerele de pe linia următoare reprezintă diferite grade de fericire în relaţia dumneavoastră. Punctul de mijloc, “fericit”, reprezintă gradul de fericire al majorităţii relaţiilor. Vă rog să încercuiţi numărul care descrie cel mai bine gradul de fericire, luând în considerare toate aspectele, ale relaţiei dumneavoastră.

| 0 | 1 | 2 | 3 | 4 | 5 | 6 |
| --- | --- | --- | --- | --- | --- | --- |
| Extrem de nefericit | Relativ nefericit | Un pic nefericit | Fericit | Foarte fericit | Extrem de fericit | Perfect |

**I32.** Vă rog să însemnaţi una dintre afirmaţiile următoare care descrie cel mai bine felul în care consideraţi viitorul relaţiei dumneavoastră.

**1.** Vreau cu disperare ca relaţia mea să reuşească şi aş face aproape orice pentru ca asta să se întâmple.

**2.** Vreau foarte tare ca relaţia mea să reuşească şi voi face tot ce pot pentru asta.

**3.** Vreau foarte tare ca relaţia mea să reuşească şi voi face ceea ce ţine de mine pentru asta.

**4.** Ar fi frumos ca relaţia mea să reuşească , dar nu pot face mai mult decât fac acum pentru asta.

**5.** Ar fi frumos ca relaţia mea să reuşească, dar refuz să mai fac ceva în plus decât fac acum pentru a face relaţia să meargă.

**6.** Relaţia mea nu poate reuşi niciodată iar eu nu mai pot face nimic pentru a face relaţia să meargă.
