# Supplementary material for: Sociological dimensions of marital satisfaction in Romania
Source: PLoS One. 2020 Aug 20;15(8):e0237923. doi: 10.1371/journal.pone.0237923 (PMC7446780; doi:10.1371/journal.pone.0237923)
Supplement: Attachment — Submitted filename: Rebuttal Letter.docx [file pone.0237923.s003.docx]

Rebuttal Letter

Sociological dimensions of marital satisfaction in Romania

Delia Nadolu, Remus Runcan and Aurel Bahnaru

Journal Requirements:

1. Please ensure that your manuscript meets PLOS ONE's style requirements, including those for file naming. The PLOS ONE style templates can be found at <https://journals.plos.org/plosone/s/file?id=wjVg/PLOSOne_formatting_sample_main_body.pdf> and <https://journals.plos.org/plosone/s/file?id=ba62/PLOSOne_formatting_sample_title_authors_affiliations.pdf>

*We have made the update of the manuscript format (first page, abstract and level 1 heading).*

2. Please include additional information regarding the survey or questionnaire used in the study and ensure that you have provided sufficient details that others could replicate the analyses. For instance, if you developed a questionnaire as part of this study and it is not under a copyright more restrictive than CC-BY, please include a copy, in both the original language and English, as Supporting Information.

*We have added the Romanian copy of the questionnaire (the English version was already included).*

3. We note you have included a table to which you do not refer in the text of your manuscript. Please ensure that you refer to Tables 2 and 3 in your text; if accepted, production will need this reference to link the reader to the Tables.

*We have corrected these aspects.*

**Comments to the Author**

1. Is the manuscript technically sound, and do the data support the conclusions?

The manuscript must describe a technically sound piece of scientific research with data that supports the conclusions. Experiments must have been conducted rigorously, with appropriate controls, replication, and sample sizes. The conclusions must be drawn appropriately based on the data presented.

Reviewer #1: Yes

Reviewer #2: Yes

2. Has the statistical analysis been performed appropriately and rigorously?

Reviewer #1: Yes

Reviewer #2: No

3. Have the authors made all data underlying the findings in their manuscript fully available?

Reviewer #1: Yes

Reviewer #2: No

*We will upload the database with English labels.*

4. Is the manuscript presented in an intelligible fashion and written in standard English?

Reviewer #1: Yes

Reviewer #2: Yes

5. Review Comments to the Author

Reviewer #1: Being from the US, I would have liked more information on marriage in general in Romania. This would have been beneficial to having a better comprehension of the findings in the study.

*In response to Reviewer 1’s request for more information on marriage in Romania we have added a chart showing statistical trends. To give wider context this is supplemented by data from other European countries. A comment on the likely reasons for the patterns shown has also been made.*

Reviewer #2: There are some things that should be done to make the data analysis clearer and more complete.

1. Sample size issues. Although there were 455 persons who responded to the survey as indicated in your methodology section, the Ns reported in the tables were substantially less. In Table 1 the highest N was 282, and in Table 3 it was only 83. There is no explanation of what became of the missing cases. This is a very high rate of missing data, and in such cases some way of handling this should be used, such as imputation, or a more complete discussion of the missing data and what bias it might yield should be included.

*The sample size issues in the data analysis arise from the composite definition of the factors analysed and the rate of participant response to specific component items in the questionnaire. This is now explained in the text of the paper.*

*The point about potential bias has been addressed in the additional paragraph that reads as follows:*

*This restriction of usefulness in our available dataset does imply some degree of limitation on the robustness of any correlations that we detected, and this consideration may be felt to apply even more to the DAS and complex factor correlations presented later. However, while maintaining a measure of caution, although we cannot claim this subset of the data fully represented our larger original sample, the surviving positive correlations remain statistically significant findings. It is arguable that, in the absence of any bias arising from the incompleteness of the responses, those relationships would have emerged more clearly than they did from a restricted sample.*

2. I have some concerns about the measure of liberalism of sexual attitudes scale. These seem to primarily to refer to views on premarital sex, but little is said about the relationship to marital sexual attitudes.

*Little has been said about the relationship between liberalism of sexual attitude and marital sexual attitude because, for reasons now more explicitly explained, this dimension was not explored in the questionnaire administered.*

As the DAS has items related to sexual satisfaction in the relationship, perhaps some additional analysis of how this relates to sexual attitudes scale would be useful.

*In response some further results of statistical analysis have been presented (pag 12). In the sense that they are negative results (no significant differences found) we are not clear how much they really add, and they would not conventionally be reported. But if they are felt to give helpful clarification we are happy to include them.*

3. Correlations of your measures with some respondent characteristics would help put the findings in context. What are the differences reported by males and females? Perhaps some differences by age and length of the relationship should also be reported here.

*Thank you very much for this recommendation. We have included an analysis of these aspects on pp 12-13.*

4. I can't see in the material I received that you will be providing access to the data set, at least the individual cases and responses of the respondents on the variables you used in this paper. This would be necessary for other researchers to explore the findings more completely using multivariate models.

*We have prepared the database with English labels and we will upload this to the system for full free access.*
